# Association between the uric acid-to-albumin ratio and risk of all-cause mortality in centenarians: a prospective cohort study

**DOI:** 10.3389/fnut.2026.1790083

**Published:** 2026-04-10

**Authors:** Xiaofei Liu, Hao Li, Qing Luo, Qiushi Wang, Zeyu Qu, Zehao Zhang, Jie Zhang, Xinye Jin, Zhe Feng, Weiguang Zhang, Qiao Zhu, Yali Zhao, Yao He, Xiangmei Chen, Ding Sun, Miao Liu, Yizhi Chen

**Affiliations:** 1 Department of Nephrology, Hainan Chen Xiangmei Academician Team Innovation Center for Kidney Diseases Research, Hainan Hospital of Chinese PLA General Hospital, Sanya, China; 2 Department of Rheumatology and Immunology, Hainan Hospital of Chinese PLA General Hospital, Sanya, China; 3 Hainan Medical University, Haikou, China; 4 Senior Department of Nephrology, State Key Laboratory of Kidney Diseases, Beijing Key Laboratory of Medical Devices and Integrated Traditional Chinese and Western Drug Development for Severe Kidney Diseases, Beijing Key Laboratory of Digital Intelligent TCM for the Prevention and Treatment of Pan-vascular Diseases, Key Disciplines of National Administration of Traditional Chinese Medicine (ZYYZDXK-2023310), National Clinical Research Center for Kidney Diseases, Chinese PLA General Hospital, Innovation Team and Talents Cultivation Program of National Administration of Traditional Chinese Medicine (ZYYCXTD-D-202402), Beijing, China; 5 Central Laboratory, Hainan Hospital of Chinese PLA General Hospital, Sanya, China; 6 Institute of Geriatrics, the Second Medical Center of Chinese PLA General Hospital, National Clinical Research Center for Geriatrics Diseases, Beijing Key Laboratory of Geriatric Comorbidity, Beijing, China; 7 Department of Anti-Nuclear, Biological, Chemical Medicine, the Graduate School of Chinese PLA General Hospital, Beijing, China; 8 The Second School of Clinical Medicine, Southern Medical University, Guangzhou, China; 9 Sanya Nephrology Medical Quality Control Center, Sanya, China

**Keywords:** albumin, all-cause mortality, centenarians, uric acid, uric acid-to-albumin ratio

## Abstract

**Background:**

The uric acid-to-albumin ratio (UAR) has emerged as a potential composite biomarker reflecting oxidative stress and nutritional status, both of which are relevant to aging and mortality risk. However, its prognostic value in extremely long-lived individuals remains unclear.

**Methods:**

A prospective cohort study involving 1,002 centenarians from China Hainan was conducted between June 2014 and December 2016. Participants were followed for survival status through March 31, 2023. Restricted cubic spline (RCS) modeling, Cox proportional hazards regression, and Kaplan–Meier survival analyses were employed to assess the association between UAR and mortality risk.

**Results:**

After excluding 78 centenarians, the cohort included 924 centenarians (median age: 102 years; 18.29% male). During a median follow-up of 29.70 months, 854 (92.42%) died. RCS analysis indicated a statistically significant overall association between UAR and mortality (adjusted *P* for overall = 0.009), with evidence of non-linearity (adjusted *P* for non-linearity = 0.029). In multivariable Cox regression analysis, individuals in the higher UAR quartile (Q4) demonstrated a 28.7% increased risk of mortality compared with those in the lower three quartiles (Q1–Q3) (adjusted hazard ratio: 1.287, 95% CI: 1.093,1.516; *P* = 0.003). Kaplan–Meier analysis further revealed that participants in Q4 had a significantly shorter median survival time (26 months) compared with those in Q1–Q3 (32 months) (log-rank test, *P* < 0.001).

**Conclusion:**

Elevated UAR is independently associated with increased all-cause mortality in centenarians, suggesting its potential utility as a prognostic biomarker for risk stratification in exceptionally long-lived populations.

## Background

Research on centenarians provides critical insights into the biological processes of aging and longevity (1). Our prior work identified hypoalbuminemia as a significant predictor of mortality in community-dwelling centenarians (2), and low serum albumin independently predicts in-hospital mortality in adults aged ≥ 90 years (3). Serum albumin is both a marker of nutritional status and an independent stratifier of mortality risk in older populations (4).

Uric acid is implicated in antiaging mechanisms (5). However, its physiological role is complex and paradoxical (6). Lower serum uric acid correlates with increased all-cause mortality (7), elevated levels raise cardiovascular (8) and metabolic diseases and associate with higher cardiovascular and overall mortality (9–11). Biomarker research in aging populations has largely focused on single indices, but uric acid and albumin both link functionally to the inflammation–oxidative stress axis (12), suggesting their interaction may influence aging-related disease processes.

The uric acid-to-albumin ratio (UAR) is a novel composite biomarker integrating metabolic, inflammatory, and nutritional dimensions to holistically assess systemic oxidative and inflammatory status (13). However, its prognostic significance in centenarian populations remains inadequately characterized. This study investigates the prognostic value of UAR for mortality risk among centenarians, aiming to refine mortality risk stratification in the oldest-old and provide empirical evidence for targeted interventions, including nutritional optimization and antioxidant therapy.

## Materials and methods

### Study design and population

This study utilized data from the China Hainan Centenarian Cohort Study (CHCCS), a prospective cohort conducted in Hainan Province between June 2014 and December 2016 (14). Among the initial cohort of 1,002 centenarians enrolled in this study, 78 participants were excluded due to missing baseline uric acid or albumin data, yielding a final analytical sample of 924 subjects. Mortality data were obtained from the National Cause of Death Registration and Reporting Information System, which is overseen by the Chinese Center for Disease Control and Prevention. To ensure data validity, reported deaths were verified through local civil affairs authorities. Additionally, the Hainan Provincial Civil Affairs Bureau conducts monthly verification of survival status for individuals aged 80 years and older to maintain the accuracy of mortality data. Ethical approval for the study was obtained from the Ethics Committee of Hainan Hospital of the Chinese People's Liberation Army General Hospital (Approval No. 301HNLL-2016-01), with all procedures conducted in strict accordance with the Declaration of Helsinki and its amendments.

### Covariates

All participants underwent detailed baseline assessments. Demographic variables collected included age, gender, ethnicity, marital status, educational attainment, smoking and alcohol consumption habits, and the presence of diabetes mellitus (DM), hypertension, and coronary heart disease (CHD). Anthropometric data were obtained through standardized measurements of weight and height, from which body mass index (BMI) was calculated as weight (kg) divided by height squared (m^2^). Blood samples were collected by trained nursing staff under standardized protocols. Albumin (bromocresol green colorimetry, g/L), uric acid (uricase-peroxidase enzymatic assay, μmol/L), and creatine (enzymatic colorimetric assay, μmol/L) were measured using a Cobas 8,000 automated analyzer (Roche Diagnostics, Basel, Switzerland) at the Central Laboratory of Hainan Hospital, Chinese People's Liberation Army General Hospital. For UAR calculation, uric acid was converted to mg/dL (μmol/L × 0.0168) and albumin to g/dL (g/L ÷ 10). The UAR was then calculated using the following formula: UAR (mg/g) = [uric acid (μmol/L) × 0.0168] / [albumin (g/L) ÷ 10] as previously described (15).

### Statistical analysis

The assumptions of normality and homogeneity of variance were evaluated prior to statistical testing. Continuous variables exhibiting a normal distribution were summarized as mean ± standard deviation (SD) and compared between groups using independent samples *t*-tests or analysis of variance (ANOVA), as appropriate. For continuous variables with non-normal distributions, data were reported as medians with interquartile ranges (IQRs), and group comparisons were conducted using the Mann–Whitney *U*-test or the Kruskal–Wallis *H*-test. Categorical variables were expressed as frequencies and percentages [n (%)] and analyzed using the chi-square test.

The association between UAR levels and all-cause mortality was examined using restricted cubic spline (RCS) regression, with UAR treated as a continuous variable. RCS models were constructed using the rms package in R software (16), with four knots assigned at the 5th, 35th, 65th, and 95th percentiles of the UAR distribution. Both unadjusted and adjusted models were applied. Prior to Cox proportional hazards regression analyses, the proportional hazards assumption was verified. Hazard ratios (HRs) and corresponding 95% confidence intervals (CIs) were estimated using univariate Cox regression. Multivariate Cox models were subsequently constructed to adjust for potential confounders, including demographic characteristics (age, gender, BMI, ethnicity, marital status, and education level), lifestyle factors (smoking status and alcohol consumption), comorbid conditions (DM, hypertension, and CHD), and serum creatinine. Time-to-event data were visualized using Kaplan–Meier survival curves, and intergroup differences were evaluated using the log-rank test. A two-tailed *p-*value < 0.05 was considered statistically significant. All analyses were conducted using R software (version 4.3.3).

## Results

### Baseline information

After 78 centenarians were excluded, 924 centenarians were enrolled in the present study (median age: 102 years; 18.29% male) (Table 1). During a median follow-up period of 29.70 months, 92.42% of participants experienced mortality. Most individuals adhered to a non-smoking (89.39%) and non-drinking (82.58%) lifestyle. The prevalence rates of hypertension, DM, and CHD were 74.13%, 9.52%, and 4.33%, respectively. The median BMI across all participants was 18.08 (16.09, 20.00) kg/m^2^. The mean albumin concentration was 38.76 ± 3.56 g/L, while the mean uric acid level was 327.34 ± 90.09 μmol/L. Participants were stratified into quartiles according to UAR values: Q1 (0.50 ≤ UAR < 1.13), Q2 (1.13 ≤ UAR < 1.39), Q3 (1.39 ≤ UAR < 1.69), and Q4 (1.69 ≤ UAR ≤ 2.94). Statistically significant differences among quartiles were observed in sex distribution (*P* < 0.001), smoking status (*P* = 0.032), prevalence of hypertension (*P* = 0.028), albumin concentrations (*P* < 0.001), uric acid levels (*P* < 0.001), and creatine levels (*P* < 0.001).

**Table 1:** Baseline characteristics of the study participants.

Variables	Overall	Q1 [0.50, 1.13]	Q2 [1.13, 1.39]	Q3 [1.39, 1.69]	Q4 [1.69, 2.94]	*P*
No. of participants	924	231	231	231	231	
Age, years	102.00 (101.00, 104.00)	102.00 (101.00, 104.00)	102.00 (101.00, 104.00)	102.00 (101.00, 104.00)	102.00 (101.00, 104.00)	0.955
Male, %	169 (18.29)	19 (8.23)	25 (10.82)	53 (22.94)	72 (31.17)	< 0.001
Follow-up time, months	29.70 (14.67, 52.78)	31.00 (15.15, 55.15)	35.00 (15.10, 57.00)	31.60 (14.80, 52.80)	26.10 (12.40, 45.60)	0.045
Death, %	854 (92.42)	208 (90.04)	206 (89.18)	215 (93.07)	225 (97.40)	0.003
Ethnicity						0.149
Han, %	818 (88.53)	209 (90.48)	208 (90.04)	206 (89.18)	195 (84.42)	
Other, %	106 (11.47)	22 (9.52)	23 (9.96)	25 (10.82)	36 (15.58)	
Marital						0.293
Separation/divorce/widow, %	828 (89.61)	205 (88.74)	213 (92.21)	209 (90.48)	201 (87.01)	
Married, %	96 (10.39)	26 (11.26)	18 (7.79)	22 (9.52)	30 (12.99)	
Education						0.197
Illiterate, %	842 (91.13)	217 (93.94)	216 (93.51)	207 (89.61)	202 (87.45)	
Elementary school, %	62 (6.71)	11 (4.76)	11 (4.76)	18 (7.79)	22 (9.52)	
Junior high school and above, %	20 (2.16)	3 (1.30)	4 (1.73)	6 (2.60)	7 (3.03)	
Smoke						0.032
Never, %	826 (89.39)	215 (93.07)	212 (91.77)	201 (87.01)	198 (85.71)	
Smoking in the past or quit, %	66 (7.14)	14 (6.06)	14 (6.06)	19 (8.23)	19 (8.23)	
Now, %	32 (3.46)	2 (0.87)	5 (2.16)	11 (4.76)	14 (6.06)	
Drinking						0.325
Never, %	763 (82.58)	199 (86.15)	192 (83.12)	192 (83.12)	180 (77.92)	
Drinking in the past or quit, %	70 (7.58)	15 (6.49)	14 (6.06)	17 (7.36)	24 (10.39)	
Now, %	91 (9.85)	17 (7.36)	25 (10.82)	22 (9.52)	27 (11.69)	
Hypertension, %	685 (74.13)	174 (75.32)	186 (80.52)	159 (68.83)	166 (71.86)	0.028
Diabetes mellitus, %	88 (9.52)	21 (9.09)	21 (9.09)	22 (9.52)	24 (10.39)	0.960
Coronary heart disease, %	40 (4.33)	8 (3.46)	12 (5.19)	7 (3.03)	13 (5.63)	0.437
Body mass index, kg/m^2^	18.08 (16.09, 20.00)	17.58 (15.91, 19.47)	18.42 (16.19, 20.31)	18.29 (16.23, 20.00)	18.21 (16.34, 20.10)	0.090
Albumin, g/L	38.76 ± 3.56	39.96 ± 3.53	39.46 ± 3.30	38.14 ± 3.40	37.45 ± 3.43	< 0.001
Uric acid, μmol/L	327.34 ± 90.09	225.48 ± 40.08	293.89 ± 31.04	346.86 ± 34.32	443.14 ± 58.53	< 0.001
Creatine, μmol/L	78.00 (65.00, 98.00)	64.00 (54.50, 75.00)	74.00 (62.50, 88.50)	85.00 (73.00, 101.00)	100.00 (80.00, 124.00)	< 0.001

Normal data: mean ± SD, compared by *t*-test or ANOVA; skewed data: median (IQR), compared by Mann–Whitney *U* or Kruskal–Wallis test; categorical data: *n*(%), compared by χ^2^ test. No., number; Q1–Q4 represent the 1st−4th quartiles of the uric acid-to-albumin ratio.

### RCS analysis of the association between UAR and all-cause mortality

**Figure 1:**
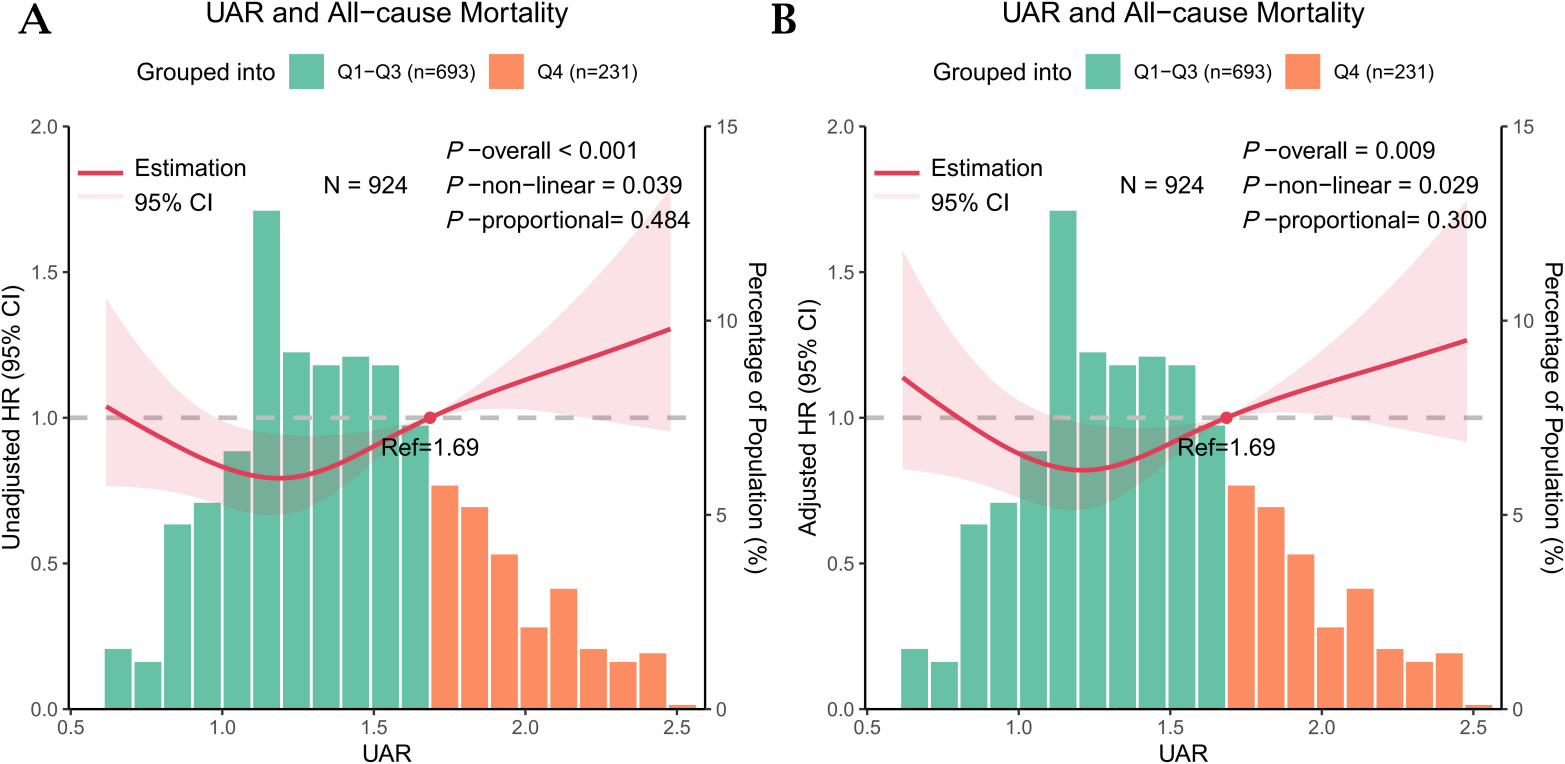
Restricted cubic spline analysis of the uric acid-to-albumin ratio (UAR) and all-cause mortality in centenarians. The solid red line indicates the hazard ratio (HR), and the shaded areas indicating 95% confidence intervals (CIs). The x-axis shows UAR levels, the left y-axis displays HRs, and the right y-axis reflects the distribution of UAR. **(A)** unadjusted model. **(B)** Adjusted model, controlling for age, gender, body mass index, ethnicity, marital status, education, smoking, alcohol consumption, diabetes, hypertension, coronary heart disease, and serum creatinine. Histograms show the population distribution according to the 75th percentile of UAR (1.69 mg/g; dashed line), with green representing Q1–Q3 and orange representing Q4. UAR was non-linearly associated with all-cause mortality (*P* overall < 0.05; *P* non-linear < 0.05). The Cox model satisfied the proportional hazards assumption (*P* > 0.05).

RCS analysis revealed a significant positive association between UAR and all-cause mortality risk in the univariate model (*P* for overall < 0.001), with evidence of non-linearity (*P* for non-linearity = 0.039; Figure 1A). After adjusting for potential confounders, the association remained significant (*P* for overall = 0.009), and the non-linear pattern was preserved (*P* for non-linearity = 0.029; Figure 1B). The RCS model confirmed UAR met the proportional hazards assumption in Cox regression, with *p*-values of 0.484 (unadjusted) and 0.300 (adjusted) for the proportionality test.

### Cox regression and Kaplan-Meier analysis between UAR and all-cause mortality

UAR was independently associated with mortality risk, whether analyzed as a continuous variable or stratified by quartiles, with the highest all-cause mortality observed in the highest quartile (Q4) (Table 2). In the unadjusted model, each 0.1-unit increase in UAR was associated with a 2.7% increase in mortality risk (HR = 1.027, 95% CI: 1.010–1.044; *P* = 0.001). This association remained significant after multivariable adjustment (HR = 1.021, 95% CI: 1.002–1.040; *P* = 0.033). Quartile-based analysis revealed that individuals in Q4 had a significantly greater mortality risk compared with those in Q2 (the lowest-hazard quartile on the RCS curve). In the unadjusted model, Q4 participants exhibited a 40.6% increased risk relative to Q2 (HR = 1.406, 95% CI: 1.163–1.700; *P* < 0.001). This risk increase was sustained after adjustment (HR = 1.359, 95% CI: 1.111–1.662; *P* = 0.003). When comparing Q4 with Q1–Q3 collectively, the unadjusted model indicated a 31.2% elevated risk (HR = 1.312, 95% CI: 1.127–1.529; *P* < 0.001), which decreased slightly in the adjusted model (HR = 1.287, 95% CI: 1.093–1.516; *P* = 0.003). A significant dose-response relationship was observed, with all models demonstrating a trend toward increased mortality risk with higher UAR levels [*P* for trend = 0.002 (unadjusted), 0.029 (adjusted)].

**Table 2:** Univariate and multivariate Cox analyses based on uric acid-to-albumin ratio (UAR).

Term	Count	Univariate analysis		Multivariate -adjusted analysis	
**UAR**	***N***	**HR**	***P***	**HR**	***P***
Continuous, per 0.1-unit	924	1.027 (1.010, 1.044)	0.001	1.021 (1.002, 1.040)	0.033
Grouped by interquartile values					
Q1 [0.50, 1.13]	231	1.070 (0.883, 1.298)	0.489	1.085 (0.892, 1.320)	0.413
Q2 [1.13, 1.39]	231	1 (Reference)		1 (Reference)	
Q3 [1.39, 1.69]	231	1.150 (0.949, 1.392)	0.153	1.082 (0.889, 1.316)	0.433
Q4 [1.69, 2.94]	231	1.406 (1.163, 1.700)	< 0.001	1.359 (1.111, 1.662)	0.003
*P* for trend			0.002		0.029
Grouped by P75 values					
Q1-Q3 [0.50, 1.69]	693	1 (Reference)		1 (Reference)	
Q4 [1.69, 2.94]	231	1.312 (1.127, 1.529)	< 0.001	1.287 (1.093, 1.516)	0.003

Q1–Q4 represent the 1st−4th quartiles of the uric acid-to-albumin ratio (UAR). Multivariate analysis was adjusted for age, gender, ethnicity, marital status, education level, body mass index, smoking status, alcohol consumption, diabetes mellitus, hypertension, coronary heart disease, and creatinine level.

**Figure 2:**
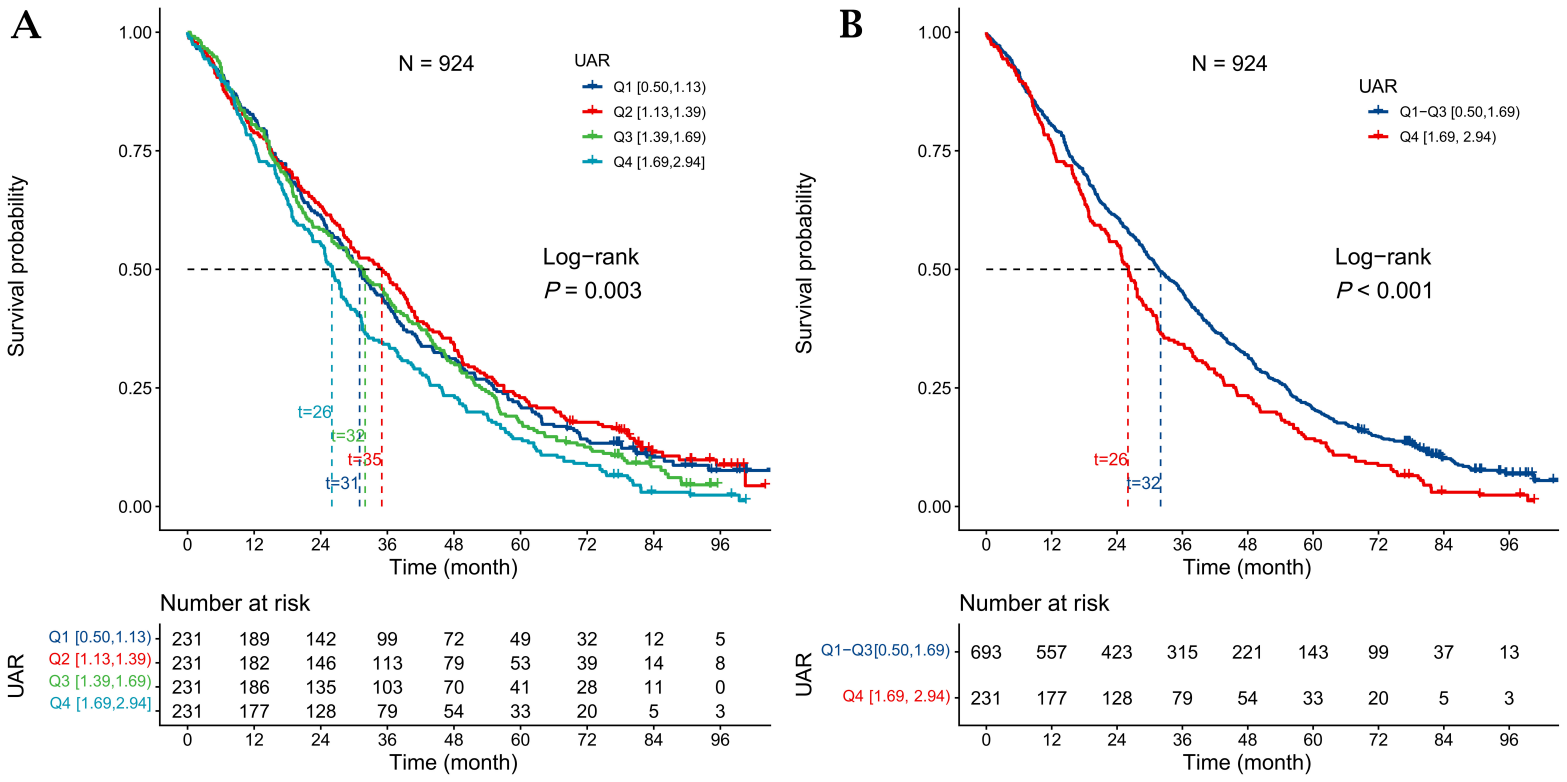
Kaplan–Meier survival curves according to the uric acid-to-albumin ratio (UAR). **(A)** Survival curves stratified by UAR quartiles; median survival was 26 months in Q4 and 35 months in Q2 (log-rank *P* = 0.003). **(B)** Survival curves comparing Q4 with Q1–Q3; median survival was 26 months in Q4 and 32 months in Q1–Q3 (log-rank *P* < 0.001).

Kaplan-Meier survival analysis further supported these findings, revealing a significant inverse correlation between UAR and survival duration. Participants in Q4 had a shorter median survival time compared with those in Q2 (26 vs. 35 months; log-rank test, *P* = 0.003; Figure 2A). Similarly, stratification using the P75 cutoff showed that individuals in the high-UAR subgroup (Q4) had reduced survival compared with those in the low-UAR subgroup (Q1–Q3) (26 vs. 32 months; log-rank test, *P* < 0.001; Figure 2B).

### Subgroup analysis of potential variables that could influence the association between UAR and all-cause mortality

**Figure 3:**
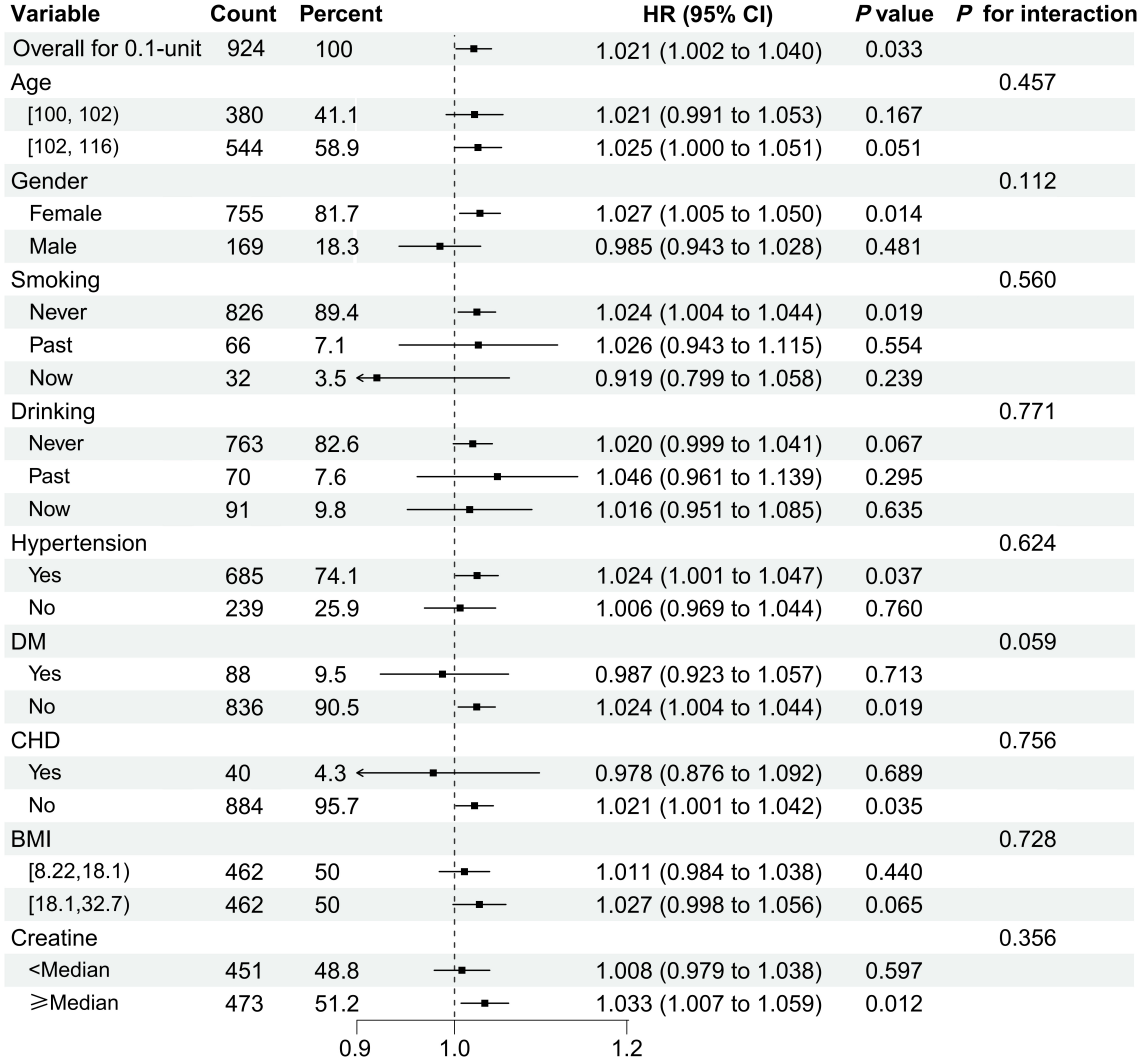
Subgroup analysis of the association between the uric acid-to-albumin ratio (UAR) and all-cause mortality. The forest plot shows adjusted hazard ratio (HR) and 95% confidence intervals (CIs) across subgroups. Multivariable models were adjusted for age, gender, body mass index, ethnicity, marital status, education, smoking, alcohol consumption, diabetes, hypertension, coronary heart disease, and serum creatinine. Each 0.1-unit increase in UAR was associated with a higher risk of all-cause mortality (adjusted HR 1.021, 95% CI: 1.002–1.040; *P* = 0.033). No significant interaction was observed across subgroups (all *P* for interaction > 0.05).

Forest plot analysis of adjusted HRs with 95% CIs confirmed the association between UAR and mortality across predefined subgroups (Figure 3). Each 0.1-unit increase in UAR was significantly associated with higher mortality risk (adjusted HR = 1.021, 95% CI: 1.002–1.040; *P* = 0.033). No significant interaction effects were observed across subgroups (all *P* for interaction > 0.05), indicating that the association between UAR and mortality was consistent across the examined strata.

## Discussion

Centenarians, who approach the maximal human lifespan with a relatively low burden of major age-related diseases, represent a valuable model for aging research. Identifying independent predictors of health outcomes in this population may help clarify aging mechanisms and inform targeted prevention. To our knowledge, this is the first large-scale prospective cohort study to investigate the association between UAR and all-cause mortality in centenarians. In this cohort of 924 centenarians, elevated UAR levels were significantly associated with increased mortality risk. UAR remained an independent predictor of mortality after adjustment for demographics, lifestyle factors, comorbidities, and serum creatinine. Subgroup analyses further supported the robustness of this association.

The association between serum uric acid and all-cause mortality has often been described as U-shaped, reflecting its dual role as both a proinflammatory mediator and an antioxidant (17). Hyperuricemia has been associated with an increased risk of hypertension (HR = 1.41, 95% CI:1.23–1.58) (18) and higher all-cause mortality in hypertensive individuals (19). Our previous work also identified a high prevalence of hyperuricemia in centenarians, which was independently associated with hypertriglyceridemia and elevated low-density lipoprotein cholesterol levels (20). In contrast, a 35-year longitudinal study showed that low baseline uric was associated with a nearly 2-fold higher likelihood of attaining centenarian status (21), whereas higher uric acid levels were associated with a reduced risk of cognitive decline in male non-agenarians and centenarians (22). These findings underscore the complex and context-dependent role of serum uric acid in mortality risk, highlighting both its potential value and its limitations as a standalone biomarker.

Albumin is a key indicator of nutritional status and systemic inflammation, with chronic inflammation being a major contributor to hypoalbuminemia (23). Serum albumin levels decline with age, particularly among individuals aged ≥ 90 years (24), and higher levels have been positively associated with survival in centenarians (25). Nevertheless, baseline albumin levels alone appear insufficient to predict centenarian status (21).

As a composite index of uric acid and albumin, UAR may better reflect oxidative stress, inflammatory burden, and nutritional status than either biomarker alone. UAR has been reported as a prognostic marker in myocardial infarction, acute pericarditis, acute myocarditis, acute kidney injury and diabetes (13, 26–29). Unlike previously studied populations with specific acute or chronic diseases, centenarians have distinctive physiological characteristics, including altered inflammatory status, oxidative stress burden, nutritional reserve, disease composition, and survival patterns. In this context, our study not only demonstrated an independent association between elevated UAR and mortality, but also identified a non-linear dose-response relationship using restricted cubic spline analysis. These results are broadly consistent with prior research suggesting that composite biomarkers improve prognostic assessment and extend the potential value of UAR to the oldest-old population.

From a clinical perspective, UAR is a simple, low-cost, and accessible biomarker derived from two routine laboratory measures, making it especially feasible in geriatric and community settings, including resource-limited areas. Although the observed effect size was modest, such markers may still be clinically meaningful in centenarians, a population in whom risk prediction is inherently challenging and effective prognostic tools remain limited. Accordingly, UAR may serve as a useful supplementary marker within routine geriatric assessment, alongside evaluation of nutritional status, inflammatory burden, comorbidity burden, and functional status. In our cohort, a UAR value above 1.69 mg/g (75th percentile) may help identify centenarians at higher mortality risk who may benefit from closer follow-up and more individualized assessment. Nevertheless, UAR should not be interpreted as a standalone predictor, but rather as part of a multidimensional risk assessment framework in very elderly individuals.

This study has several strengths, including its prospective design, a relatively large sample of centenarians, and a median follow-up of 29.7 months. Notably, this is the first large-scale cohort study to examine the relationship between UAR and mortality risk specifically in centenarians. Our findings extend the potential clinical relevance of UAR beyond disease-specific settings and suggest its utility as a practical prognostic biomarker in the oldest-old population, in whom effective risk stratification tools remain limited. However, several limitations should be acknowledged. First, the study population comprised exclusively Chinese centenarians, which may restrict the generalizability of findings to other ethnic groups. Second, the analysis was limited to all-cause mortality, precluding the association of UAR with specific causes of death. Further large-scale, multi-ethnic cohort studies with more complete clinical data and cause-of-death information are needed to validate and extend our findings.

## Conclusion

This study helps fill an important gap in research on composite biomarkers for mortality risk stratification in the oldest-old and provides empirical evidence supporting the clinical relevance of UAR in centenarians, a population for whom effective prognostic biomarkers remain limited. Our findings demonstrate that elevated UAR is significantly and independently associated with increased all-cause mortality among centenarians, suggesting that UAR may serve as a novel, low-cost and accessible independent supplementary biomarker for mortality risk in this population.

## Data Availability

The raw data supporting the conclusions of this article will be made available by the authors, without undue reservation.
